# Artificial intelligence approaches to predicting treatment non-adherence in chronic diseases: a narrative review

**DOI:** 10.3389/fdgth.2026.1769337

**Published:** 2026-04-14

**Authors:** Sharmake Gaiye Bashir, Hiba Abdi Salad, Yakub Burhan Abdullahi, Yusuf Hared Abdi, Mohamed Sharif Abdi, Naima Ibrahim Ahmed, Shuaibu Saidu Musa

**Affiliations:** 1 Faculty of Health Sciences and Tropical Medicine, Somali National University, Mogadishu, Somalia; 2 Research Unit, Center of Excellence, Somali National University, Mogadishu, Somalia; 3 Department of Nursing Science, Ahmadu Bello University, Zaria, Nigeria; 4 Faculty of Medicine, School of Global Health, Chulalongkorn University, Bangkok, Thailand

**Keywords:** artificial intelligence, chronic diseases, machine learning, medication adherence, treatment non-adherence

## Abstract

Medication non-adherence affects 40%–50% of chronic disease patients globally, causing preventable morbidity and substantial healthcare costs. Traditional adherence monitoring approaches are retrospective and reactive, limiting timely intervention. Artificial intelligence and machine learning offer novel approaches for prospective adherence risk prediction, enabling anticipatory, resource-efficient interventions. This narrative review synthesizes current evidence on AI-based non-adherence prediction across chronic diseases including HIV, tuberculosis, diabetes, hypertension, and mental health disorders. Machine learning models integrating heterogeneous data sources electronic health records, pharmacy refill patterns, sociodemographic variables, and healthcare utilization achieve discrimination metrics (AUC 0.70–0.95) superior to traditional risk stratification. These AUC values are reported descriptively to reflect model discrimination within individual studies and should not be interpreted as results of formal comparison or quantitative synthesis across diseases or modeling approaches. However, significant barriers constrain clinical translation: limited external validation, algorithmic bias affecting marginalized populations, inadequate interpretability, data privacy concerns, and substantial implementation challenges in resource-limited health systems. Future research priorities include rigorous multicenter external validation, model development in low- and middle-income countries, advancement of interpretable architectures, and prospective randomized trials evaluating clinical outcomes. Responsible AI deployment requires participatory governance, health equity prioritization, and maintenance of clinician oversight throughout implementation. This review critically evaluates AI potential while emphasizing prerequisites for equitable, ethical, and clinically meaningful adherence prediction in global health contexts.

## Introduction

Chronic non-communicable diseases (NCDs) constitute a fundamental challenge to global health, accounting for approximately 75% of worldwide mortality while imposing unprecedented economic burdens on health systems and societies (1, 2). The World Health Organization estimates that approximately 300 million people globally live with chronic obstructive pulmonary disease alone, with similar or higher prevalence rates documented for hypertension, diabetes, cardiovascular disease, and other long-term conditions (2). Although modern pharmacotherapy has transformed the therapeutic landscape for managing these conditions, the clinical and epidemiological benefits of evidence-based medications remain substantially unrealized due to treatment non-adherence (1, 3). Medication non-adherence affects between 40% and 50% of patients with chronic diseases in developed countries, with even higher proportions reported in resource-limited settings (1). This persistent challenge is estimated to cause at least 100,000 preventable deaths and USD $100 billion in avoidable medical costs annually in the United States alone, figures that substantially underestimate the global health and economic toll when considered across low- and middle-income countries where the chronic disease burden disproportionately affects vulnerable populations (1, 2).

Treatment non-adherence, however, is fundamentally a systemic and health system challenge that extends far beyond individual patient behavior or motivation (1, 4). Non-adherence emerges from the complex interplay of the social determinants of health, structural barriers to care, healthcare system limitations, disease-related factors, and therapy-related characteristics (4, 5). Evidence increasingly demonstrates that medication non-adherence is shaped by factors spanning multiple ecological levels: individual socioeconomic status, access to medications, health literacy, and therapeutic burden; healthcare team and system-related factors, including provider-patient communication and treatment complexity; and broader structural determinants, such as poverty, stigma, and health system fragmentation (4, 6). Traditional clinical approaches have predominantly framed non-adherence as a patient-centered problem, which is attributed to poor adherence to willful non-compliance or cognitive limitations (1, 7). This reductionist perspective has inadvertently shifted responsibility to patients while obscuring the health system deficiencies and structural inequities that fundamentally constrain treatment adherence capacity, particularly among marginalized and underserved populations (4, 8).

Contemporary adherence assessment methodologies, which are important for research and clinical monitoring, have substantial limitations that constrain their predictive utility and clinical applicability (9, 10). Self-reported adherence measures and patient-reported outcome instruments are subject to social desirability bias, recall errors, and poor specificity for identifying truly non-adherent individuals (9, 11). Pharmacy claims-based measures, including the proportion of days covered (PDC) and medication possession ratio (MPR), operationalize adherence by dispensing records rather than actual medication use, thereby assuming that filled prescriptions equate to medication intake, an assumption that introduces systematic upward bias in adherence estimates, particularly when measurements span short time frames (12, 13). Direct observational approaches and biological assessments remain resource-intensive and impractical for population-level implementations (10). More critically, traditional adherence monitoring approaches are fundamentally retrospective and reactive, capturing adherence behavior after it has occurred rather than prospectively identifying patients at imminent risk of future non-adherence (1, 14). This temporal limitation substantially constrains the capacity of health systems to implement timely and anticipatory interventions during critical windows of therapeutic vulnerability (15).

Artificial intelligence, particularly machine learning and predictive modeling methodologies, offers a fundamentally different approach to adherence assessment by enabling the synthesis and analysis of heterogeneous data sources, including electronic health records, pharmacy dispensing patterns, clinical encounters, social determinants of health indicators, and patient-reported information to generate prospective predictions of individual non-adherence risk (14). Unlike traditional monitoring approaches, AI-based predictive systems operate within anticipatory rather than reactive paradigms, potentially enabling health systems to identify patients progressing toward non-adherence before clinical deterioration occurs, and to target interventions toward populations most likely to benefit (1). Machine learning models have demonstrated promising discrimination and calibration performance across diverse clinical contexts, including hypertension, diabetes, human immunodeficiency virus, hepatitis C virus, and antiretroviral therapy, with reported accuracy metrics ranging from 70% to 91% depending on the algorithms, feature selection strategies, and validation cohorts (16, 17). Despite these developments, the clinical translation of AI-based adherence prediction remains nascent, with significant gaps in model generalizability, interpretability, algorithmic bias, data privacy, regulatory frameworks, and ethical deployment in resource-constrained healthcare systems (18, 19).

This narrative review synthesizes current evidence regarding artificial intelligence approaches used to predict treatment non-adherence in chronic diseases, with particular emphasis on methodological approaches, data sources and integration strategies, applications across disease contexts, ethical and equity considerations, regulatory dimensions, and implications for health system implementation. By critically discussing both the promises and limitations of AI-based adherence prediction, this review aims to advance the understanding of how predictive analytics can augment health system capacity to anticipate and address treatment non-adherence while maintaining equitable, patient-centered, and ethically defensible approaches to technology implementation in global health contexts.

## Methodology

### Study design

This study was conducted as a narrative review to synthesize and critically examine published evidence on artificial intelligence (AI) and machine-learning approaches for predicting treatment non-adherence in chronic diseases. A narrative review design was selected to allow flexible integration of heterogeneous evidence across disease contexts, data sources, modeling strategies, and implementation settings. The objective was to provide a conceptually informed and critical overview of current methodological trends, limitations, and future directions, rather than to perform a systematic or quantitative synthesis of evidence.

### Literature search strategy

A structured literature search was conducted across major biomedical and interdisciplinary databases, including PubMed/MEDLINE, Scopus, Web of Science, and Google Scholar. Searches primarily targeted studies published between January 2018 and December 2025, reflecting the period of rapid expansion in AI and machine-learning applications in healthcare. Search terms and combinations included “*artificial intelligence*,” “*machine learning*,” “*deep learning*,” “*predictive modeling*,” “*medication adherence*,” “*treatment non-adherence*,” and “*chronic disease*.” In addition, reference lists of relevant review articles and key empirical studies were screened to identify additional pertinent publications.

### Eligibility criteria

Studies were included if they:

(i) examined AI- or machine-learning–based models designed to predict medication or treatment non-adherence;
(ii) focused on chronic diseases or long-term treatment contexts; and
(iii) were published as full, peer-reviewed articles in English.

Studies were excluded if they focused exclusively on adherence interventions without a predictive component, consisted of editorials, commentaries, conference abstracts without full manuscripts, study protocols without results, or non–peer-reviewed sources.

### Study selection and synthesis

Study selection was guided by thematic relevance and methodological contribution, consistent with the narrative review approach. No formal quality scoring or risk-of-bias assessment was applied. Included studies were synthesized qualitatively and organized thematically according to disease context, data sources, modeling approaches, performance reporting practices, ethical and equity considerations, and health system implementation challenges.

## Methodological limitations of the review

As a narrative review, this study does not aim to provide exhaustive coverage of all available literature, nor does it apply systematic review or meta-analytic procedures. Study inclusion was informed by relevance and conceptual value, which may introduce selection bias. Nevertheless, this approach enables critical integration of methodological, ethical, and implementation-focused insights that are not readily captured through quantitative synthesis alone.

## Conceptualizing treatment non-adherence in chronic care

Treatment non-adherence in chronic disease management encompasses the degree to which a patient's medication-taking behaviors align with the prescribed therapeutic recommendations provided by healthcare providers (1, 20). The World Health Organization (WHO) defines medication adherence as “the process by which patients take their medications as prescribed,” recognizing adherence as a dynamic and multidimensional construct rather than a binary outcome (1, 21). Contemporary conceptualizations divide adherence into three interrelated phases: initiation, implementation, and persistence (21). Initiation occurs when a patient obtains and takes the first dose of a prescribed medication, which represents the extent to which actual medication intake corresponds to the prescribed dosing regimen over time, and persistence describes the duration between therapy initiation and its discontinuation (21). This tripartite framework enables granular characterization of where non-adherence occurs throughout the treatment trajectory (1, 21).

Distinguishing between primary and secondary non-adherence clarifies important operational differences with distinct clinical and public health implications (22, 23). Primary non-adherence refers to patients who never fill or initiate prescribed medications, whereas secondary non-adherence describes suboptimal medication use after treatment has been initiated, including missed doses, reduced frequency, or premature discontinuation (22, 23). Primary non-adherence remains historically understudied relative to secondary adherence and persistence; however, approximately 20% of patients with newly prescribed medications never initiate therapy, representing a substantial but often invisible barrier to treatment efficacy (22, 24). This distinction carries specific relevance for predictive modeling, as early identification of primary non-adherence risk before therapy initiation enables health systems to implement earlier support interventions, while secondary non-adherence prediction identifies maintenance-phase support opportunities (25).

Treatment non-adherence must be distinguished from other related but conceptually distinct healthcare behaviors (20, 21). Missed medical appointments and clinic non-attendance constitute separate dimensions of healthcare engagement that intersect, but are not synonymous with, medication adherence (26). Similarly, treatment interruption or discontinuation describes cessation of therapy, which may result from intentional patient decisions or unintentional lapses, yet requires different analytic and intervention approaches (27, 28). Adherence heterogeneity extends beyond these definitional distinctions to encompass substantial variability in operational measurements and classification standards across clinical research and practice settings (27, 28). Some studies employed strict thresholds (medication possession ratio ≥80%), while others used continuous adherence scales or qualitative categorizations (9, 29). This heterogeneity in definitional and operational frameworks creates significant challenges for comparability across studies and for developing standardized predictive models, as different adherence definitions may identify fundamentally different patient subgroups and generate divergent prevalence estimates (22).

The temporal limitations of retrospective adherence detection fundamentally constrain the capacity of the health system to take preventive action (1, 30). Traditional adherence assessment methods capture adherence behavior after it has occurred, identifying nonadherent patients only when clinical consequences have already materialized, including disease exacerbations, treatment failures, or poor clinical outcomes (31, 32). Pharmacy claims-based retrospective adherence measurement provides statistical snapshots of historical adherence patterns over defined time windows but offers minimal prospective predictive value regarding which currently adherent patients will transition to future non-adherence or which newly initiated patients will remain adherent during critical early implementation phases (9, 32). Early adherence patterns during the first 3 months of therapy demonstrated modest predictive accuracy for subsequent long-term adherence, with early consistent non-adherence indicating future non-adherence more reliably than initial adherence episodes (32, 33). However, this predictive window remains insufficient for timely system-level intervention (1, 30).

Machine learning and artificial intelligence approaches fundamentally invert this temporal paradigm by enabling the prospective identification of patients progressing toward future non-adherence before clinical deterioration occurs (14). Predictive risk stratification models trained on heterogeneous data sources, including demographic characteristics, clinical comorbidities, prior medication histories, pharmacy refill patterns, clinical encounter frequencies, and increasingly, social determinants of health indicators, generate forward-looking risk scores, identifying which patients will discontinue therapy, miss doses, or require support (31). These anticipatory approaches enable targeted allocation of health system resources toward high-risk populations during critical windows of therapeutic vulnerability before adherence failure progresses to clinical harm (31). The clinical utility of such predictive systems depends fundamentally upon standardized, reproducible definitions of the adherence outcome being predicted. However, operational definition heterogeneity across modeling contexts remains substantial and unresolved, limiting model transferability across health systems and clinical populations (31).

## Overview of artificial intelligence approaches for predicting non-adherence

Artificial intelligence approaches to predicting medication non-adherence encompass diverse computational methodologies that share a unifying characteristic: the capacity to identify complex, multidimensional patterns within high-dimensional healthcare data that exceed the human cognitive capacity to process intuitively (34, 35). Machine learning represents a broader conceptual framework wherein computer systems learn probabilistic relationships between input features and predicted outcomes through iterative pattern recognition, rather than following explicitly programmed decision rules (35, 36). Traditional statistical approaches estimate the parameters underlying assumed functional relationships between variables, whereas machine learning algorithms discover these relationships empirically from data, making minimal assumptions about underlying distributions or functional forms (36, 37). This fundamentally different epistemological approach enables machine learning to accommodate complex, nonlinear interactions among variables, such as the simultaneous effects of socioeconomic status, clinical comorbidity, medication complexity, and healthcare system factors that traditional linear regression models struggle to capture (35, 38).

Deep learning constitutes a specialized machine learning subclass that utilizes hierarchical, interconnected layers of computational nodes to extract increasingly abstract features from raw data through successive transformations (35). Recurrent neural networks (RNNs) and their specialized variants, including long short-term memory (LSTM) networks, represent particularly valuable deep learning architectures for medication adherence prediction because they process sequential, temporal data while maintaining the memory of prior patterns throughout extended time horizons (39). Unlike conventional machine learning approaches that treat each observation as statistically independent, temporal deep learning models explicitly encode adherence trajectory capturing when patients transition from consistent medication-taking to missed doses, whether discontinuation patterns follow identifiable precursor sequences, or when early adherence behaviors predict future persistence (39). LSTM models trained on pharmacy refill sequences and clinical encounter histories have demonstrated superior predictive accuracy for identifying future non-adherence compared to non-temporal approaches, achieving discrimination metrics (area under the curve, 0.75–0.88) substantially superior to baseline risk stratification (39, 40).

Beyond pattern detection at the individual level, machine learning enables risk stratification at the population level by generating individualized non-adherence risk scores that quantify the relative likelihood of future non-adherence heterogeneously across diverse subpopulations (41). This probabilistic risk stratification fundamentally differs from binary classification approaches (adherent vs. nonadherent) by acknowledging the inherent uncertainty in adherence prediction and enabling graduated resource allocation proportional to the predicted risk magnitude (41). Healthcare systems can deploy intervention resources toward the highest-risk quintiles, reserving intensive support for patients with predicted non-adherence probability exceeding specified thresholds, thereby optimizing finite intervention capacity (41). Risk stratification models integrate heterogeneous data sources that incorporate prior medication histories, clinical comorbidities, pharmacy refill patterns, ambulatory healthcare utilization, demographic characteristics, and increasingly, social determinants of health indicators into unified predictive frameworks that would be computationally infeasible for manual analysis (39).

Despite substantial promise, significant limitations constrain current AI applications to predict medication adherence in clinical settings (18, 42). Model generalizability represents a critical challenge, whereby algorithms developed and validated within single health systems demonstrate performance degradation when applied prospectively to different patient populations, healthcare delivery contexts, or geographic regions (42, 43). External validation testing model performance on completely independent patient cohorts not involved in model development remains inconsistently performed across adherence prediction literature, with many published models lacking rigorous external validation (42, 44). The interpretability or “explainability” challenge reflects widespread concern that complex machine learning models, particularly deep learning architectures, operate as “black boxes” providing risk scores without clinically meaningful explanations of which patient characteristics or historical patterns most strongly influenced predictions (42, 44). Healthcare providers require a transparent understanding of why specific patients receive high non-adherence risk scores to implement appropriately targeted interventions, yet complex models often generate predictions that are mathematically accurate but clinically opaque (42, 45). Heterogeneity in training data quality, completeness of clinical information, variable adherence outcome definitions, and temporal drift, wherein patient populations and clinical practices evolve over time, causing model performance deterioration, create additional barriers to reliable real-world deployment (42).

## Data sources and predictors used in AI-based non-adherence models

Electronic health records (EHRs) constitute the primary data source for training AI-based adherence prediction models, containing longitudinal clinical information including diagnostic codes, laboratory results, vital signs, medication orders, comorbidity indices, healthcare utilization patterns, and provider encounter histories (35). EHR-derived features enable the comprehensive characterization of patient clinical trajectories spanning multiple years, capturing disease progression, treatment complexity, polypharmacy burden, and cumulative healthcare engagement that collectively inform future adherence risk (46, 47), as shown in Table 1. Diagnostic history extracted from standardized coding systems, particularly International Classification of Diseases (ICD) codes across multiple clinical encounters, provides latent representations of disease co-occurrence patterns and comorbidity networks that substantially improve predictive accuracy compared with models trained on demographic variables alone (48, 49). Laboratory measurements, including glycemic control markers, lipid panels, renal function indicators, and biomarkers of disease progression, encode objective clinical status independent of patient self-report, whereas medication order histories reveal prior adherence patterns, therapeutic failures, medication switches, and regimen complexity (35, 49).

**Table 1:** Data sources and common predictors used in AI models for predicting treatment non-adherence.

Data source	Examples of predictors	Type of non-adherence predicted	Strengths	Key limitations	Reference
Electronic health records (EHRs)	Diagnostic codes (ICD), laboratory values (HbA1c, lipid panels, renal function), vital signs, medication order history, comorbidity indices, prior treatment outcomes, disease severity scores	Primary and secondary non-adherence; regimen complexity-driven non-adherence	Comprehensive longitudinal data; objective clinical measurements; capture disease progression and treatment complexity; widely available in clinical settings; standardized coding systems enable comparability	Missing values in laboratory measurements (5%–40% depending on variable type); data entry variability; temporal drift in documentation practices; does not capture actual medication consumption; restricted to documented encounters	(46–49)
Pharmacy refill data	Proportion of days covered (PDC), medication possession ratio (MPR), refill gaps, time between fills, prescription refill timing patterns, early refills, medication stockpiling behaviors, refill consistency trajectories	Secondary and persistent non-adherence; discontinuation risk	Objective, automatically captured from administrative systems; temporal granularity enables identification of refill gap patterns; historically strong predictive performance; directly reflects prescription-filling behavior; identifies primary non-adherence when integrated with e-prescribing data	Does not confirm actual medication ingestion (filled ≠ taken); systematic upward bias in adherence estimates; incomplete capture when patients fill prescriptions outside healthcare system; high cost and proprietary barriers; variable adherence definitions across studies reduce comparability	(39, 41, 50, 54, 60)
Sociodemographic variables and social determinants of health (SDOH)	Age, sex, race, ethnicity, insurance status, income level, neighborhood socioeconomic status (SES), area deprivation indices, educational attainment, employment status, housing stability, food security, transportation access, geographic distance to pharmacy, rural vs. urban residence	Primary non-adherence (initiation phase); secondary non-adherence driven by structural barriers; health system fragmentation–related non-adherence	Captures structural determinants of adherence; enables identification of vulnerable populations; supports health equity analysis; actionable targets for intervention design; increasingly available from public health databases and geospatial sources	Aggregate neighborhood-level indicators do not necessarily reflect individual circumstances; potential for racial bias if used without fairness mitigation; proxies for unmeasured social factors; privacy concerns regarding data collection and linkage; limited availability in some healthcare systems	(5, 55, 57)
Healthcare utilization and appointment data	Appointment attendance rate, no-show frequency, appointment scheduling patterns, emergency department visits, hospitalization frequency, intensive care unit admission, inpatient length of stay, continuity of care index, outpatient visit frequency, time since last provider encounter	Secondary non-adherence; treatment discontinuation; healthcare disengagement–related non-adherence	Captures healthcare engagement patterns independent of medication-taking; easily extracted from administrative systems; temporal patterns identify transitions to non-adherence; correlates with clinical outcomes	Confounded by clinical severity (sicker patients may have more appointments); does not directly measure adherence; missing data when patients receive care outside captured health systems; influenced by access barriers unrelated to adherence motivation; high correlation with multiple other non-adherence predictors	(56, 63)
Wearable devices and sensor data	Physical activity levels, sleep duration and quality, heart rate variability, respiratory rate, blood oxygen saturation, daily step counts, medication container sensor readings, smart inhaler actuations, pill bottle opening timestamps, real-time vital signs, activity rhythms	Real-time secondary non-adherence; dose-timing non-adherence; medication technique adherence (inhalers)	Real-time, continuous measurement of adherence behavior; objective data collection minimizing recall bias; enables early detection of adherence transitions; captures medication technique and timing; high temporal resolution for identifying patterns	Limited adoption in routine clinical practice; high cost and patient burden; data security and privacy concerns; incomplete population coverage (biased toward affluent, tech-literate patients); variable integration with EHR systems; high battery dependency and maintenance requirements; potential for technical failures and data gaps	(64–67)
Clinical comorbidity and treatment complexity data	Number of concurrent medications (pill burden), medication regimen complexity index, number of daily doses, frequency of dosing changes, presence of comorbid mental health conditions (depression, anxiety), chronic kidney disease stage, diabetes duration, cardiovascular comorbidity count, polypharmacy burden	Regimen complexity–driven non-adherence; disease-specific non-adherence; cognitive burden–related non-adherence	Directly captures known barriers to adherence (medication burden, regimen complexity); objective clinical data; strong association with non-adherence in observational studies; modifiable through deprescribing and regimen simplification	Comorbidity coding completeness varies; does not account for patient perception of burden; indirect relationship with adherence (complexity correlates with but does not cause non-adherence); confounded by clinical severity; missing data on medication side effects from EHR codes	(63, 68, 69)
Integrated multi-source data	Combined features from EHRs + pharmacy claims + appointment records + SDOH variables + comorbidity data + demographic characteristics + prior adherence history	Primary and secondary non-adherence; heterogeneous non-adherence phenotypes; future discontinuation risk; adherence trajectory transitions	Captures multilevel determinants of adherence (patient, provider, system, societal); enables superior discrimination compared to single-source models; reflects real-world complexity of non-adherence; enables development of risk stratification algorithms for precision intervention allocation	Substantial data fragmentation across healthcare systems reduces completeness; missing data mechanisms (missing not at random) introduce bias; increased computational complexity and hyperparameter tuning requirements; limited external validation across diverse healthcare contexts; significant privacy, security, and regulatory compliance challenges; heterogeneous data quality across sources impedes model transferability	(47, 48, 70)

Pharmacy refill data derived from administrative claims or dispensing databases provide valuable temporal information for adherence prediction models (41, 50). The proportion of days covered (PDC) and medication possession ratio (MPR) calculated retrospectively from refill sequences serve simultaneously as outcome measures and predictive features, encoding historical adherence trajectories that demonstrate temporal autocorrelation past non-adherence and predict future non-adherence (41, 50). Refill gap patterns, medication stockpiling behaviors evidenced through early refills, prescription abandonment following initial fills, and temporal dynamics of refill consistency provide granular information regarding patient medication-taking behaviors that are unavailable through other data sources (50, 51). Integration of e-prescription data with pharmacy claims enables differentiation between primary non-adherence (prescriptions written but never filled) and secondary non-adherence (prescriptions filled but consumed suboptimally), a clinically meaningful distinction with divergent intervention implications (41, 52). Pharmacy data additionally capture medication formulation switches, therapeutic substitutions, and dose adjustments that reflect the evolving treatment complexity and regimen burden (53, 54).

Sociodemographic variables and social determinants of health (SDOH) data are increasingly featured as essential predictors in adherence models, reflecting the recognition that non-adherence emerges from multilevel factors beyond clinical characteristics (5). Age, sex, race, ethnicity, insurance status, geographic location, neighborhood-level socioeconomic indicators, and area deprivation indices encode the structural determinants of healthcare access, medication affordability, health literacy, and systemic barriers to adherence (55). Appointment attendance records, healthcare utilization frequency, emergency department visits, hospitalization histories, and care continuity measures capture healthcare engagement patterns that correlate with medication adherence, while representing conceptually distinct behavioral dimensions (56). Distance to pharmacy, transportation access, rural versus urban residence, and healthcare system fragmentation operationalized through counts of distinct healthcare facilities visited represent actionable SDOH features that health systems can potentially modify through intervention design (57).

Despite the richness of available data sources, substantial data quality challenges constrain the development and deployment of real-world models (58, 59). Missing data pervades EHR systems, with laboratory values, vital signs, sociodemographic fields, and SDOH variables frequently incomplete or absent, creating systematic bias when missingness patterns correlate with adherence outcomes a scenario termed missing not at random (59). Data completeness varies dramatically across healthcare settings, patient subpopulations, and temporal periods, with reported missingness ranging from 5% to 40%, depending on variable types and data sources (58, 59). Healthcare data fragmentation resulting from patients receiving care across multiple unlinked health systems produces incomplete medication histories, unobserved clinical encounters, and artificially inflated non-adherence estimates when pharmacy fills occurring outside the captured systems remain undetected (60, 61). The gradual evolution of temporal drift in clinical documentation practices, coding standards, medication formularies, and patient population characteristics causes model performance degradation over time, as training data becomes progressively less representative of current clinical contexts (61). Standardization challenges across data sources, heterogeneous EHR systems, variable data entry quality, and lack of interoperability create additional barriers to developing generalizable and transferable prediction models applicable across diverse healthcare delivery contexts (62).

## Applications of AI-based non-adherence prediction across chronic diseases

HIV management has emerged as a leading context for developing and validating AI-based adherence prediction models, reflecting the critical importance of sustained antiretroviral therapy (ART) adherence for viral suppression and prevention of treatment-resistant strains (16). Machine learning models developed in low-resource sub-Saharan African settings have identified loss to follow-up risk, treatment interruption, and secondary non-adherence using routine electronic medical records, with random forest algorithms achieving discrimination accuracies exceeding 80% (16, 71). Key adherence predictors in HIV populations encompass clinical characteristics, including CD4 count, viral load suppression status, tuberculosis co-infection status, and health system factors, such as differentiated service delivery models and appointment-scheduling patterns (71). The integration of enhanced language models with HIV clinic narratives from Tanzania revealed that predictive insights regarding disengagement could be generated with clinical transparency, enabling the interpretation of modifiable risk factors that drive future non-adherence (72), as also shown in Table 2.

**Table 2:** Applications of AI-based non-adherence prediction across chronic diseases.

Chronic disease	Predicted outcome (non-adherence type)	AI Approach (general)	Primary data source	Reported use case	Reference
HIV/AIDS	Loss to follow-up from care; treatment interruption; secondary non-adherence to ART	Random forest, Extreme Gradient Boosting (XGBoost), Logistic regression, Support Vector Machine	Electronic medical records (EMR), CD4 count, viral load, ART refill histories, appointment attendance records	Prediction of future interruption in ART among PLWH in Nigeria with 81% sensitivity, 88% specificity; identification of loss to follow-up risk in low-resource Ethiopian settings with 84.2% accuracy; differentiated service delivery models and adherence patterns integrated into risk stratification.	(16, 71)
Tuberculosis (TB)	Treatment non-compliance during TB treatment course; loss to follow-up; treatment failure in MDR-TB	Logistic regression-based ML, XGBoost, Random Forest, Support Vector Machine, Multimodal deep learning (LSTM, CNN)	Electronic health records, chest imaging (x-rays, CT scans), pathogen genomics (drug susceptibility), clinical variables, adherence trajectories, adverse event burden	Prediction of MDR-TB treatment non-compliance with education level, registration group, treatment support, and khat use as key predictors; multimodal deep learning integrating clinical, imaging, and genomic data achieved 81%–83% accuracy in MDR-TB treatment outcome prediction; XGBoost for loss-to-follow-up prediction (AUC 0.921 pre-treatment, 0.825 in-treatment).	(73–75, 90)
Type 2 diabetes	Secondary non-adherence to glucose-lowering medications; treatment abandonment during maintenance therapy; glycemic control failure	Random Forest, Ensemble learning methods, Gradient Boosting, Support Vector Machine, Logistic Regression	Electronic health records, prior medication refill data, HbA1c trajectories, fasting blood glucose measurements, medication possession ratio (MPR), demographic characteristics, comorbidity data	ML models trained on diverse diabetes populations achieved AUC discrimination of 0.866 in predicting adherence risk; stratification into glycemia trajectory clusters (stable adequate, improving inadequate, fluctuating inadequate) with 85% balanced accuracy; key predictors included prior glucose control, disease duration, insulin vs. oral medication use.	(77, 78, 91, 92)
Hypertension	Secondary non-adherence to antihypertensive medications; uncontrolled blood pressure status; persistent treatment failure	XGBoost, Random Forest, Machine learning ensemble methods, Logistic Regression	Electronic health records, baseline blood pressure measurements, medication refill data, ambulatory blood pressure monitoring, demographic variables, comorbidity burden, physical activity levels	ML models successfully predicted uncontrolled blood pressure status within 12-month timeframes; feature importance analysis identified antihypertensive medication combinations, baseline BP severity, age, BMI, and comorbidity burden as key drivers; XGBoost demonstrated superior discriminative ability for personalized treatment recommendations.	(80, 82, 83)
Schizophrenia & bipolar disorder	Medication non-compliance to antipsychotic therapy; treatment interruption; long-term non-adherence; treatment resistance in first-episode psychosis	Machine learning-based nomograms, Bayesian probabilistic classifiers, Random Forest, Logistic Regression, Ensemble learning methods	Electronic health records, mental health forum discussions, Drug Attitude Inventory scores, Brief Psychiatric Rating Scale symptom assessments, hospitalization histories, antipsychotic use patterns, symptom variability trajectories	Nomogram prediction models identified low insight, high symptom burden, multiple hospitalizations, and prior long-acting injectable experience as independent non-compliance risk factors; Bayesian classifiers trained on mental health forum text data predicted psychiatric medication nonadherence with clinically meaningful sensitivity and specificity; ML frameworks for first-episode psychosis identified relapse frequency, symptom trajectories, and baseline diagnosis as key predictors of treatment resistance.	(85, 86, 89)

Tuberculosis treatment, particularly multidrug-resistant tuberculosis (MDR-TB), represents another disease context in which AI-based adherence prediction demonstrates substantial clinical value as treatment interruption directly enables the emergence of further drug resistance (73, 74). Machine learning models integrating clinical health records, imaging data, and pathogen genomics have achieved 81% accuracy in predicting MDR-TB treatment outcomes, with key predictors including medication adherence patterns, treatment regimen composition, and adverse event burden (73, 75). Multimodal deep learning approaches incorporating the temporal dynamics of clinical variables, imaging findings, and adherence trajectories have outperformed models using static clinical features alone, reflecting the value of capturing disease progression patterns during treatment (39, 73). Logistic regression-based machine learning algorithms have successfully identified MDR-TB patients at imminent risk of non-compliance during their treatment course, enabling early targeted adherence support before treatment failure occurs (74, 76).

Type 2 diabetes management increasingly incorporates AI adherence prediction frameworks focused on identifying patients likely to abandon glucose-lowering medications or experience secondary non-adherence during maintenance therapy (31, 77). Machine learning models trained on diverse diabetes populations achieved an area-under-curve discrimination of 0.866 in predicting adherence risk with important predictors including prior medication adherence patterns, HbA1c trajectory clusters, and insulin utilization status (31, 78). Prediction models have successfully stratified heterogeneous diabetes patient cohorts into long-term glycemic trajectory classes (stable adequate, improving but inadequate, fluctuating inadequate) with a balanced accuracy of 85%, enabling the identification of patients requiring intensified metabolic management or adherence support (31, 78). Feature importance analysis revealed that prior glucose control, disease duration, insulin vs. oral medication use, and monotherapy vs. combination therapy were the key drivers of future adherence trajectories (31, 78).

Hypertension presents unique adherence prediction challenges given the asymptomatic nature of the disease and the high burden of polypharmacy, which requires complex regimens (79, 80). Machine learning models using routine EHR data have successfully predicted uncontrolled blood pressure status within 12-month timeframes with clinically actionable accuracy, enabling the identification of patients requiring treatment intensification or adherence support before blood pressure remains uncontrolled (80). Feature importance analysis revealed that antihypertensive medication combinations, baseline blood pressure severity, age, body mass index, physical activity levels, and comorbidity burden were key drivers of blood pressure control success, indicating selective targeting of adherence interventions toward medication combinations most likely to fail in specific patient subgroups (81, 82). Personalized treatment recommendations derived from machine learning approaches offer the potential to optimize medication selection and dosing, while accounting for individual response heterogeneity (83, 84).

Mental health disorders, particularly schizophrenia and bipolar disorder, exhibit exceptionally high medication non-adherence rates (30%–50%), driven by cognitive impairment, psychotic symptoms, medication side effects, and illness-related factors (85, 86). Machine learning-based nomograms predicting antipsychotic medication adherence identified low insight (Drug Attitude Inventory scores), high symptom burden (Brief Psychiatric Rating Scale), multiple hospitalizations, and prior experience with long-acting injectable formulations as independent risk factors for noncompliance (85, 87). Probabilistic Bayesian machine learning classifiers trained on mental health forum discussions successfully predicted medication nonadherence among psychiatric patients with clinically meaningful sensitivity and specificity, offering scalable approaches to identify at-risk populations using cost-effective data sources (86). Machine learning frameworks for predicting treatment resistance in first-episode psychosis identified baseline diagnosis, age at psychosis onset, symptom variability trajectories, relapse frequency, and antipsychotic use patterns as key longitudinal predictors, suggesting that early adherence patterns during critical therapeutic windows substantially influenced long-term treatment outcomes (88, 89).

## Model performance, interpretability and clinical utility

The predictive performance of AI-based adherence models has been reported across multiple, sometimes inconsistent, metrics in the published literature, creating challenges for comparing models across studies (93). Area under the receiver operating characteristic curve (AUC-ROC) remains the most frequently reported discrimination metric, with reported values ranging from 0.70 to 0.95 depending on disease context, feature selection strategies, and validation cohorts (93). Sensitivity and specificity describe classification accuracy at chosen probability thresholds, whereas positive and negative predictive values provide context-specific estimates relevant to particular patient populations (93). The reported accuracy metrics typically range from 75% to 90% across adherence prediction studies, although accuracy alone provides limited clinical insight regarding the types of errors (false positives vs. false negatives) that occur most frequently (93, 94). Calibration assessment evaluating whether predicted probabilities align with observed adherence outcomes remains inconsistently reported despite the substantial importance for clinical decision-making, as poorly calibrated models may mislead clinicians regarding actual patient risk (93). Beyond these traditional discrimination and calibration metrics, decision curve analysis has emerged as a superior framework for evaluating clinical utility, plotting net benefits across variable decision thresholds to quantify the clinical value of adherence predictions for different treatment scenarios (95).

The interpretability and explainability of complex AI models constitute critical barriers to clinical adoption, as clinicians require a transparent understanding of which patient characteristics drive a high adherence risk score (96). Shapley Additive explanations (SHAP) and Local Interpretable Model-agnostic Explanations (LIME) have become increasingly adopted methods for *post-hoc* model interpretation, decomposing complex model predictions into individual feature contributions, enabling clinicians to understand why specific patients receive high-risk stratification (97). Feature importance analysis frequently identifies disease-modifiable factors, such as medication regimen complexity, appointment scheduling patterns, and treatment side effect burden, alongside non-modifiable demographic characteristics, supporting the actionability of predictions (98, 99). However, competing demands between predictive accuracy and interpretability remain unresolved, as simpler, more interpretable models (logistic regression, decision trees) frequently demonstrate inferior discrimination compared to complex ensemble methods or deep learning approaches that operate as intractable “black boxes.” (96) This accuracy-interpretability tradeoff fundamentally constrains clinical implementation, as healthcare systems must balance superior discrimination, potentially enabling better patient identification against the transparency required for provider trust and accountability (96, 100).

Clinical actionability, the capacity of predictions to inform specific, implementable clinical decisions, extends beyond discrimination and interpretability to encompass workflow feasibility and implementation context (96, 101). A model demonstrating 85% AUC yet requiring data unavailable at point-of-care or necessitating complex probabilistic interpretations, clinicians cannot readily translate to action, offers minimal clinical utility despite strong statistical performance (96, 101). Conversely, simpler models generating interpretable risk scores that enable obvious, resource-efficient interventions may provide superior net clinical benefits, despite lower discrimination metrics (95, 96). Decision curve analysis explicitly quantifies this net benefit across clinically relevant probability thresholds, enabling stakeholders to evaluate whether the proposed interventions would improve outcomes compared to treat-all or treat-none strategies (95). Contemporary literature increasingly emphasizes that discrimination metrics alone, even when supplemented by calibration and interpretability assessment, insufficiently characterize clinical utility without explicit evaluation of implementation feasibility, cost-effectiveness relative to existing workflows, and potential harms from false-positive stratification, unnecessarily flagging compliant patients for intensive intervention (96). Achieving clinical utility requires explicit dialogue between model developers, clinicians, health system stakeholders, and patients to ensure that AI-based adherence predictions are integrated seamlessly into existing workflows while generating actionable insights that physicians can confidently implement (96, 102).

## Methodological quality challenges in AI-based non-adherence prediction studies

Despite promising discrimination performance, several methodological limitations constrain current AI-based non-adherence prediction models. Overfitting is common, particularly in studies developed on small or single-center datasets, limiting generalizability. External validation using independent cohorts is inconsistently performed, reducing confidence in model transportability across settings and populations. Dataset imbalance, where non-adherence events are relatively infrequent, may inflate reported AUC values while masking poor performance among high-risk patients. In addition, reliance on retrospective electronic health record and pharmacy data introduces bias due to missing or incomplete information, particularly for socially marginalized populations. Heterogeneity in non-adherence definitions across studies further complicates performance interpretation. Importantly, few models have been prospectively evaluated within real-world clinical workflows, highlighting the gap between methodological performance and clinical applicability.

**Figure 1:**
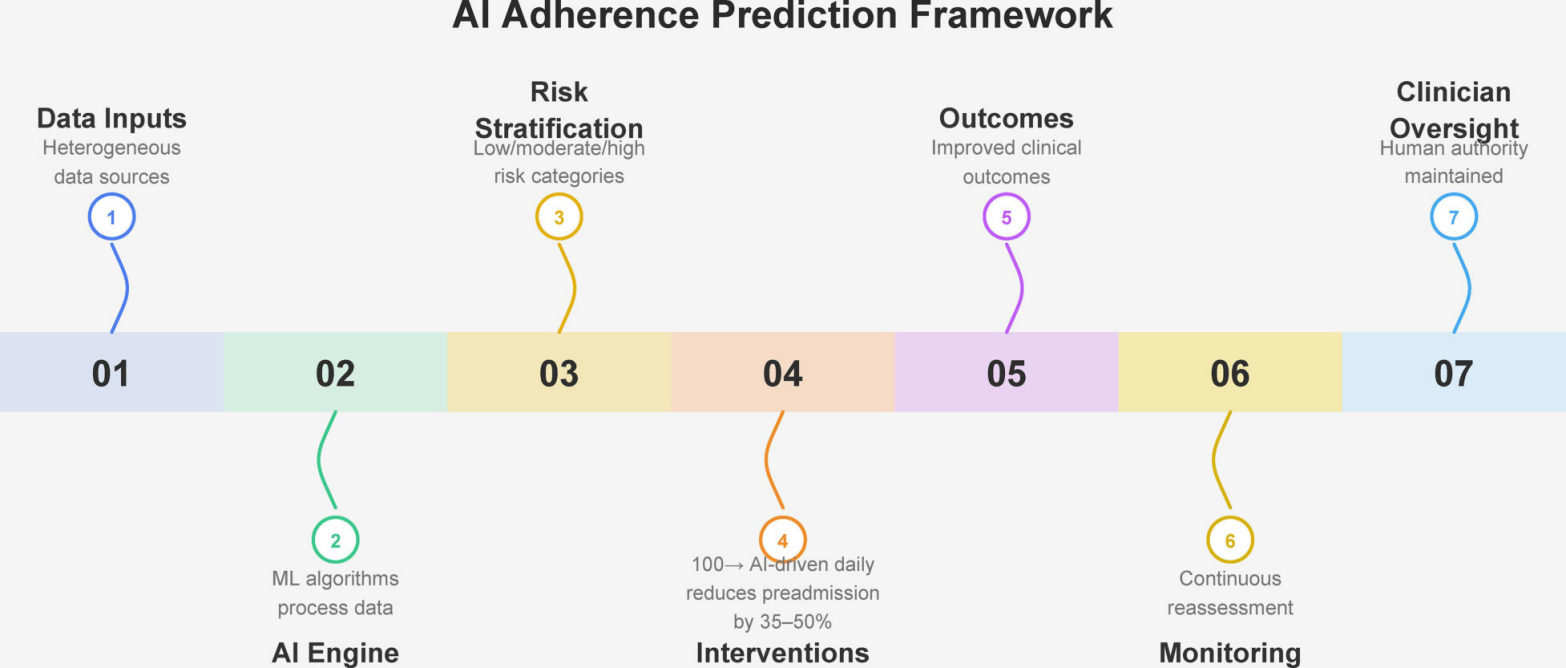
Conceptual framework for artificial intelligence-based prediction and risk-stratified intervention for treatment non-adherence in chronic diseases.

Figure 1 illustrates a sequential AI-driven framework in which heterogeneous patient data are processed by machine learning models to predict treatment non-adherence risk, stratify patients into risk categories, and guide targeted clinical interventions. Continuous monitoring and clinician oversight ensure iterative refinement of predictions and support improved adherence and clinical outcomes across populations with chronic diseases.

## Ethical, equity, and governance considerations

Algorithmic bias represents a fundamental ethical challenge in AI-based adherence prediction, as models trained on unrepresentative data may systematically underperform in racial, ethnic, and socioeconomically disadvantaged populations, thereby perpetuating or amplifying existing health disparities (19). Biases emerge through multiple pathways: underrepresentation of minority groups in training datasets, failure to account for social determinants of health that differ across populations, and the incorporation of proxy variables encoding historical discrimination (19, 103). Without explicit fairness evaluation and mitigation strategies, high-performing models may achieve excellent overall discrimination while simultaneously generating disparate prediction errors across demographic groups, with false-positive rates systematically higher for marginalized populations (19, 104). Fairness-aware algorithms, demographic parity assessment, and equalized odds evaluation must accompany all model developments to ensure that adherence risk predictions translate equitably across diverse patient populations (19, 105).

Privacy and consent considerations present acute ethical tensions in adherence prediction contexts, as these models require the integration of sensitive personal data on medication-taking behaviors, mental health status, and social circumstances (106, 107). Patients may lack awareness of how their data are utilized for algorithmic prediction, particularly in low-resource settings where written informed consent mechanisms remain limited (107, 108). Data governance structures protecting patient rights remain inadequately specified in most low- and middle-income countries, creating vulnerability to unauthorized data re-identification, secondary use without consent, and breaches compromising patient confidentiality (107, 108). The ethical imperative requires the implementation of transparent data governance frameworks to ensure meaningful patient consent, explicit data use limitations, and accountability mechanisms that respond to patient concerns (106, 107).

Stigmatization represents a distinct ethical concern in mental health contexts, where non-adherence predictions generated from algorithmic models may reinforce harmful stereotypes regarding psychiatric patients’ untrustworthiness or unreliability, thereby amplifying the existing discrimination in clinical care and employment (86, 109). The algorithmic identification of high non-adherence risk in mental health populations must be accompanied by explicit safeguards to prevent predictive insights from being weaponized for punitive policies or resource rationing (106, 107). Health systems must prioritize the development of adherence prediction models in resource-limited and fragile settings to prevent the widening of the global digital divide in precision health technologies (107, 110). Without intentional capacity building in low-resource regions, adherence prediction innovations will be concentrated in high-income healthcare systems, exacerbating global health inequities (107, 111). The implementation of AI-based adherence prediction requires participatory governance structures involving patients, frontline health workers, and community representatives in algorithm development, validation, and deployment decision-making (107, 112). Governance frameworks must explicitly specify accountability mechanisms, transparency requirements, and ongoing algorithmic auditing to ensure that adherence predictions remain equitable, non-discriminatory, and aligned with health system equity goals throughout operational deployment (106, 107).

## Implementation challenges and health system readiness

The implementation of AI-based adherence prediction models in routine clinical practice faces substantial infrastructure barriers that extend beyond model development to encompass interoperability, data quality, computational capacity, and technical sustainability (45, 113). Most health systems operate fragmented EHR platforms lacking standardized data structures, preventing the seamless integration of adherence prediction algorithms into clinical workflows (45). Health systems report critical gaps in technical infrastructure, including unreliable Internet connectivity, inadequate computational resources, and lack of cybersecurity frameworks necessary for secure deployment of predictive models, particularly in low-resource regions (45, 113). Real-world implementation requires a transition from validation on curated datasets to continuous operation with live clinical data characterized by incompleteness, measurement error, and temporal drift, requiring ongoing model monitoring and retraining (45, 114). These infrastructure challenges necessitate substantial upfront capital investment and sustained operational resources that are often unavailable in underresourced health systems (45, 113).

Workforce capacity and clinician training represent critical implementation barriers, as healthcare providers frequently lack familiarity with machine learning concepts, model interpretation, and the appropriate application of algorithmic risk scores in clinical decision-making (115). Training requirements span not only end-user clinicians, but also data managers, IT specialists, and health system leaders requiring specialized knowledge in Machine Learning Healthcare Operations (MLHOps) for model deployment and maintenance (45). Clinicians’ trust in AI-based predictions remains fragile without transparent interpretable models grounded in clinical logic recognizable to providers (45, 116). Studies examining clinician perspectives reveal that healthcare providers reject ML solutions lacking clear clinical validation and interpretable decision-making processes, and demonstrate superior discrimination compared to existing clinical judgment (45). Implementation success requires substantial investment in workforce education, user-centered design of clinical interfaces, and organizational change management processes that enable clinician adoption (45, 117).

Clinical workflow integration presents distinct implementation challenges, as adherence prediction algorithms must be seamlessly incorporated into existing care processes without imposing excessive cognitive burden or disrupting clinical efficiency (45, 114). Poorly designed clinical decision support systems generate alert fatigue through excessive notifications, prompting clinicians to ignore genuine risk stratification and undermine model utility (45, 114). Successful implementation requires iterative co-design with frontline clinicians defining acceptable model behavior, optimal alert timing and frequency, and intervention pathways that enable actionable clinical responses (45, 117). Organizational sustainability demands the establishment of governance structures that specify accountability for model performance, processes for continuous quality monitoring, and mechanisms for updating algorithms as patient populations and clinical practices evolve (45, 113). Health system commitment to AI implementation requires alignment with institutional strategic priorities and competitive pressure to adopt these technologies, creating tension between innovation diffusion and the realistic assessment of implementation capacity and clinical utility (45, 113).

**Figure 2:**
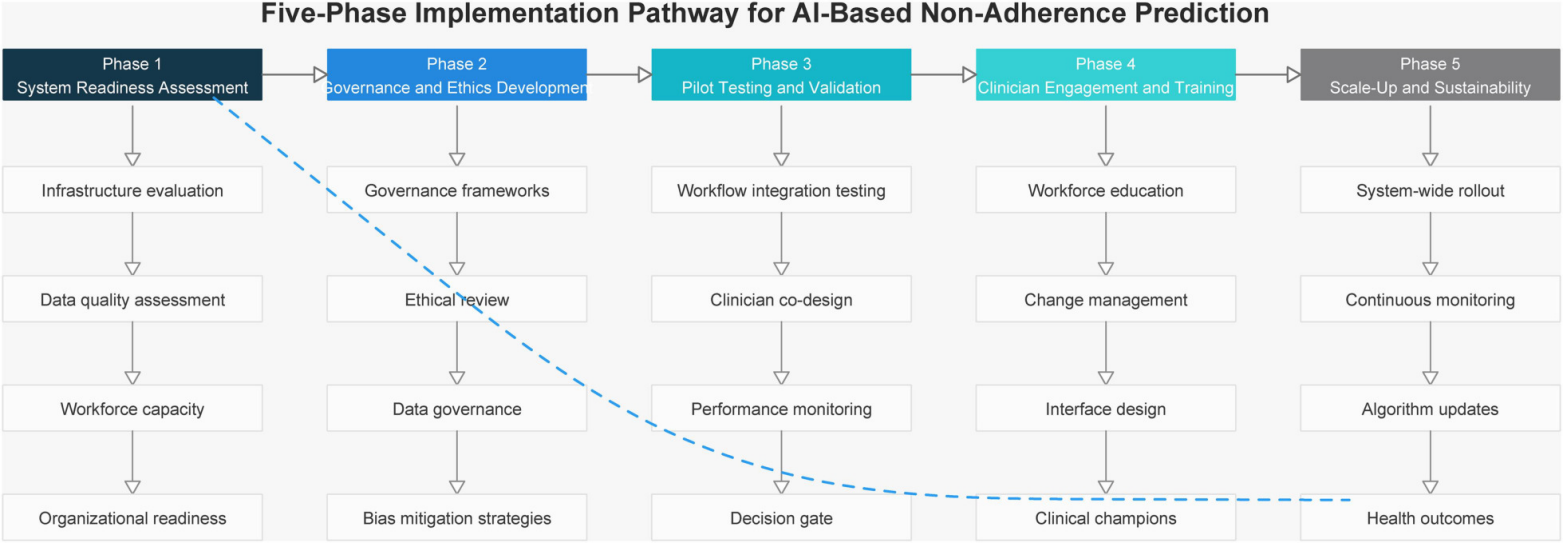
Five-phase implementation pathway for integrating AI-based prediction of treatment non-adherence into health systems.

Figure 2 presents a five-phase pathway for integrating AI-based prediction of treatment non-adherence to health systems. The framework progresses from system readiness and governance foundations through pilot testing and clinician co-design to workforce engagement and sustainable scale-up, supported by continuous monitoring and iterative learning.

## Future directions and research priorities

Rigorous external validation across diverse healthcare systems, geographic regions, and patient populations is the most critical methodological priority for advancing AI-based adherence prediction from development to clinical deployment (44, 118). Contemporary literature demonstrates that models achieving strong internal validation frequently exhibit substantial performance degradation when applied to external cohorts, with AUC reductions of 0.05–0.15 common across multicenter validation studies (119, 120). Establishing standardized validation frameworks enabling rapid assessment across international data networks, as demonstrated by the OHDSI collaborative infrastructure, could dramatically accelerate generalizability evaluation while reducing the typical 3-year validation timeline (44, 121). Development and prospective evaluation of adherence prediction models in low- and middle-income countries remain critically underrepresented, despite these regions bearing a disproportionate chronic disease burden and experiencing the greatest potential benefit from resource-efficient AI-based risk stratification (107, 122). Research priorities must include participatory model development with LMIC stakeholders, validation using locally collected data, and explicit assessment of implementation feasibility given the infrastructure constraints characteristic of resource-limited settings (107, 108). Advancing interpretability beyond *post-hoc* explanation methods toward inherently interpretable model architectures represents an essential research direction, as clinician trust and regulatory approval increasingly demand transparent decision-making processes, rather than black-box predictions supplemented with SHAP values (42, 96). Prospective randomized controlled trials evaluating clinical outcomes, cost-effectiveness, and implementation processes remain scarce, with most adherence prediction literature terminating at model development rather than demonstrating the actual impact on patient adherence or clinical disease control (44, 113).

## Contributions of this article

- Provides an updated synthesis of current evidence on the application of artificial intelligence (AI) technologies in chronic disease management, particularly in clinical decision-making, patient monitoring, and personalized treatment strategies.
- Offers a broader perspective compared with previous reviews by examining multiple AI approaches across different chronic conditions rather than focusing on a single disease or specific AI technique.
- Highlights emerging opportunities and practical challenges in implementing AI within real-world healthcare systems, including issues related to data quality, ethical considerations, and clinical integration.
- Identifies key gaps in the existing literature and outlines areas for future research to improve the development and adoption of AI-driven tools for chronic disease management.

## Study limitations

Several limitations should be considered when interpreting the findings of this review. First, this study was conducted as a narrative review rather than a systematic review or meta-analysis, which may limit the comprehensiveness and reproducibility of the literature selection process. Although a structured search strategy was applied across major databases, relevant studies published in other databases, grey literature, or non-English sources may not have been captured. Second, the included studies demonstrate considerable heterogeneity in terms of disease contexts, data sources, model architectures, and adherence outcome definitions, which constrains direct comparison of model performance across studies. Third, many AI-based adherence prediction models reported in the literature rely on retrospective electronic health record or administrative datasets, which may contain incomplete or missing data and may not fully capture real-world medication-taking behaviors. Finally, relatively few studies have evaluated these models prospectively in routine clinical workflows, highlighting the gap between methodological development and real-world implementation.

## Conclusion

Artificial intelligence approaches for predicting treatment non-adherence represent a promising, yet nascent field offering substantial potential to advance personalized, anticipatory interventions targeting the global adherence challenge. Machine learning models capable of synthesizing heterogeneous longitudinal patient data to generate prospective non-adherence risk predictions could enable health systems to allocate limited intervention resources to populations most likely to benefit, potentially improving medication adherence rates and clinical disease control across diverse chronic diseases. However, this potential remains substantially unrealized, contingent upon addressing critical methodological, organizational, and ethical barriers spanning external validation across healthcare contexts, demonstration of clinical impact through rigorous prospective trials, and explicit commitment to equitable implementation in resource-limited settings bearing a disproportionate chronic disease burden. Real-world deployment experiences reveal that AI implementation success requires simultaneous attention to technical performance, human factors, organizational readiness, governance oversight, and sustained clinician engagement, and not merely superior model discrimination. Health systems and policymakers must adopt measured, evidence-based approaches to AI integration that preserve clinical judgment, maintain human oversight, and prioritize health equity throughout the implementation lifecycle. The responsible development and deployment of AI-based adherence prediction models, rooted in participatory governance and validated across diverse populations, offer a realistic pathway toward augmenting rather than replacing clinical decision-making to advance global treatment adherence and improve chronic disease outcomes.

## References

[1] Kardas P Bennett B Borah B Burnier M Daly C Hiligsmann M Medication non-adherence: reflecting on two decades since WHO adherence report and setting goals for the next twenty years. Front Pharmacol. (2024) 15:1444012. 10.3389/FPHAR.2024.1444012 39764461 PMC11700791

[2] Hacker K. The burden of chronic disease. Mayo Clin Proc Innov Qual Outcomes. (2024) 8:112. 10.1016/J.MAYOCPIQO.2023.08.005 38304166 PMC10830426

[3] Kleinsinger F. The unmet challenge of medication nonadherence. Perm J. (2018) 22:18–33. 10.7812/TPP/18-033 30005722 PMC6045499

[4] Leddy AM Jaganath D Triasih R Wobudeya E Bellotti De Oliveira MC Sheremeta Y Social determinants of adherence to treatment for tuberculosis infection and disease among children, adolescents, and young adults: a narrative review. J Pediatric Infect Dis Soc. (2022) 11:S79–84. 10.1093/JPIDS/PIAC058 36314549 PMC9620428

[5] Donneyong MM Chang TJ Jackson JW Langston MA Juarez PD Sealy-Jefferson S Structural and social determinants of health factors associated with county-level variation in non-adherence to antihypertensive medication treatment. Int J Environ Res Public Health. (2020) 17:6684. 10.3390/IJERPH17186684 32937852 PMC7557537

[6] Ferreira PD Jr Simoes JA Sr Velho DC Sr. Adherence to antihypertensive therapy and its determinants: a systematic review. Cureus. (2024) 16:e59532. 10.7759/CUREUS.59532 38826951 PMC11144025

[7] Krousel-Wood M Craig LS Peacock E Zlotnick E O’Connell S Bradford D Medication adherence: expanding the conceptual framework. Am J Hypertens. (2021) 34:895. 10.1093/AJH/HPAB046 33693474 PMC8457429

[8] Augusta TL Howard S Mary A. Exploring hypertension medication adherence in African Americans using the health belief model and the social determinants of health. Res Theory Nurs Pract. (2025) 39:151–68. 10.1891/RTNP-2024-0071 40588416

[9] Fontanet CP Choudhry NK Isaac T Sequist TD Gopalakrishnan C Gagne JJ Comparison of measures of medication adherence from pharmacy dispensing and insurer claims data. Health Serv Res. (2022) 57:524–36. 10.1111/1475-6773.13714;ISSUE:ISSUE:DOI 34387355 PMC9108079

[10] Bohlmann A Mostafa J Kumar M. Machine learning and medication adherence: scoping review. JMIRX Med. (2021) 2:e26993. 10.2196/26993 37725549 PMC10414315

[11] Glassberg MB Trygstad T Wei D Robinson T Farley JF. Accuracy of prescription claims data in identifying truly nonadherent patients. J Manag Care Spec Pharm. (2019) 25:1349–56. 10.18553/JMCP.2019.25.12.1349 31778616 PMC10398018

[12] Vollmer WM Xu M Feldstein A Smith D Waterbury A Rand C. Comparison of pharmacy-based measures of medication adherence. BMC Health Serv Res. (2012) 12:155. 10.1186/1472-6963-12-155 22691240 PMC3413584

[13] Maciejewski ML Bryson CL Wang V Perkins M Liu CF. Potential bias in medication adherence studies of prevalent users. Health Serv Res. (2013) 48:1468. 10.1111/1475-6773.12043 23402554 PMC3725535

[14] Ghozali MT. Predicting patient adherence in healthcare using artificial intelligence and machine learning techniques: a narrative review. In 8th International Conference on I-SMAC (IoT in Social, Mobile, Analytics and Cloud), I-SMAC 2024. (2024). p. 1211–5.

[15] Reis ZSN Pereira GMV Dias CdS Lage EM de Oliveira IJR Pagano AS. Artificial intelligence-based tools for patient support to enhance medication adherence: a focused review. Front Digit Health. (2025) 7:1523070. 10.3389/FDGTH.2025.1523070/BIBTEX 40364851 PMC12069381

[16] Yeneakal KA Teferi GH Mihret TT Mengistu AK Tizie SB Tadele MM. Predicting antiretroviral therapy adherence status of adult HIV-positive patients using machine-learning northwest, Ethiopia, 2025. BMC Med Inform Decis Mak. (2025) 25:259. 10.1186/S12911-025-03106-4 40640758 PMC12247312

[17] Marineci CD Valeanu A Chiriță C Negreș S Stoicescu C Chioncel V. Development and validation of predictive models for non-adherence to antihypertensive medication. Medicina. (2025) 61:1313. 10.3390/MEDICINA61071313 40731942 PMC12300708

[18] Liang Z Suresh A Chen IY. Revealing treatment non-adherence bias in clinical machine learning using large language models (2025).

[19] Sufian MA Alsadder L Hamzi W Zaman S Sagar ASMS Hamzi B. Mitigating algorithmic bias in AI-driven cardiovascular imaging for fairer diagnostics. Diagnostics. (2024) 14:2675. 10.3390/DIAGNOSTICS14232675 39682584 PMC11640708

[20] Chapman SCE Chan AHY. Medication nonadherence - definition, measurement, prevalence, and causes: reflecting on the past 20 years and looking forwards. Front Pharmacol. (2025) 16:1465059. 10.3389/FPHAR.2025.1465059/BIBTEX 40124783 PMC11925869

[21] Vrijens B De Geest S Hughes DA Przemyslaw K Demonceau J Ruppar T A new taxonomy for describing and defining adherence to medications. Br J Clin Pharmacol. (2012) 73:691. 10.1111/J.1365-2125.2012.04167.X 22486599 PMC3403197

[22] Adams AJ Stolpe SF. Defining and measuring primary medication nonadherence: development of a quality measure. J Manag Care Spec Pharm. (2016) 22:516–23. 10.18553/JMCP.2016.22.5.516 27123913 PMC10398291

[23] Zeitouny S Cheng L Wong ST Tadrous M McGrail K Law MR. Prevalence and predictors of primary nonadherence to medications prescribed in primary care. Can Med Assoc J. (2023) 195:E1000–9. 10.1503/CMAJ.221018/TAB-RELATED-CONTENT 37553145 PMC10446155

[24] Korb-Savoldelli V Tran Y Perrin G Touchard J Pastre J Borowik A Psychometric properties of a machine learning–based patient-reported outcome measure on medication adherence: single-center, cross-sectional, observational study. J Med Internet Res. (2023) 25:e42384. 10.2196/42384 37843891 PMC10616746

[25] Başaranoğlu M Taşdemir İK Akbay E Doruk HE. Artificial intelligence-based prediction of treatment failure and medication non-adherence in overactive bladder management. BMC Urol. (2025) 25:209. 10.1186/S12894-025-01911-7 40841657 PMC12369125

[26] Wang Y Feng Z Ying X Peiyu W Liu N. Frequency, barriers and facilitators of adherence to treatment among people with systemic lupus erythematosus in China: a scoping review. BMJ Open. (2025) 15:e091845. 10.1136/BMJOPEN-2024-091845 40829832 PMC12366575

[27] Halpin DMG Rothnie KJ Banks V Czira A Compton C Wood R Comparative adherence and persistence of single- and multiple-inhaler triple therapies among patients with chronic obstructive pulmonary disease in an English real-world primary care setting. Int J Chron Obstruct Pulmon Dis. (2022) 17:2417–29. 10.2147/COPD.S370540 36185170 PMC9519012

[28] Labauge P Créange A Moreau T Nouvet-Gire J Pedespan B Heinzlef O TEC-ADHERE: real-world persistence and adherence on dimethyl fumarate in patients with relapsing–remitting multiple sclerosis in the French OroSEP patient-support program. Neurol Ther. (2024) 14:177–92. 10.1007/S40120-024-00674-X 39527163 PMC11762029

[29] De Geest S Zullig LL Dunbar-Jacob J Hughes D Wilson IB Vrijens B. Improving medication adherence research reporting: ESPACOMP medication adherence reporting guideline (EMERGE). Eur J Cardiovasc Nurs. (2019) 18:258. 10.1177/1474515119830298 30739497 PMC6433491

[30] Kumamaru H Lee MP Choudhry NK Dong YH Krumme AA Khan N Using previous medication adherence to predict future adherence. J Manag Care Spec Pharm. (2018) 24:1146–55. 10.18553/JMCP.2018.24.11.1146 30362915 PMC10397923

[31] Wu X-W Yang H-B Yuan R Long E-W Tong R-S. Predictive models of medication non-adherence risks of patients with T2D based on multiple machine learning algorithms. BMJ Open Diab Res Care. (2020) 8:1055. 10.1136/bmjdrc-2019-001055 PMC7064141 32156739

[32] Franklin JM Shrank WH Lii J Krumme AK Matlin OS Brennan TA Observing versus predicting: initial patterns of filling predict long-term adherence more accurately than high-dimensional modeling techniques. Health Serv Res. (2015) 51:220. 10.1111/1475-6773.12310 25879372 PMC4722199

[33] Alhazami M Pontinha VM Patterson JA Holdford DA. Medication adherence trajectories: a systematic literature review. J Manag Care Spec Pharm. (2020) 26:1138–52. 10.18553/jmcp.2020.26.9.1138 32857646 PMC10391275

[34] Liu H Tripathy RK. Machine learning and deep learning for healthcare data processing and analyzing: towards data-driven decision-making and precise medicine. Diagnostics. (2025) 15:1051. 10.3390/DIAGNOSTICS15081051 40310409 PMC12025807

[35] Rahman A Debnath T Kundu D Khan MSI Aishi AA Sazzad S Machine learning and deep learning-based approach in smart healthcare: recent advances, applications, challenges and opportunities. AIMS Public Health. (2024) 11:58. 10.3934/PUBLICHEALTH.2024004 38617415 PMC11007421

[36] Habehh H Gohel S. Machine learning in healthcare. Curr Genomics. (2021) 22:291. 10.2174/1389202922666210705124359 35273459 PMC8822225

[37] Pettit RW Fullem R Cheng C Amos CI. Artificial intelligence, machine learning, and deep learning for clinical outcome prediction. Emerg Top Life Sci. (2021) 5:729. 10.1042/ETLS20210246 34927670 PMC8786279

[38] Rani P Kumar R Jain A Lamba R Sachdeva RK Kumar K An extensive review of machine learning and deep learning techniques on heart disease classification and prediction. Arch Comput Methods Eng. (2024) 31:3331–49. 10.1007/S11831-024-10075-W

[39] Hsu W Warren JR Riddle PJ. Medication adherence prediction through temporal modelling in cardiovascular disease management. BMC Med Inform Decis Mak. (2022) 22:313. 10.1186/S12911-022-02052-9 36447245 PMC9710081

[40] Gu Y Zalkikar A Liu M Kelly L Hall A Daly K Predicting medication adherence using ensemble learning and deep learning models with large scale healthcare data. Sci Rep. (2021) 11:18961. 10.1038/S41598-021-98387-W 34556746 PMC8460813

[41] Zullig LL Jazowski SA Wang TY Hellkamp A Wojdyla D Thomas L Novel application of approaches to predicting medication adherence using medical claims data. Health Serv Res. (2019) 54:1255. 10.1111/1475-6773.13200 31429471 PMC6863234

[42] Ennab M Mcheick H. Enhancing interpretability and accuracy of AI models in healthcare: a comprehensive review on challenges and future directions. Front Robot AI. (2024) 11:1444763. 10.3389/FROBT.2024.1444763 39677978 PMC11638409

[43] Goetz L Seedat N Vandersluis R van der Schaar M. Generalization—a key challenge for responsible AI in patient-facing clinical applications. NPJ Digit Med. (2024) 7:126. 10.1038/S41746-024-01127-3 38773304 PMC11109198

[44] Yuan H. Toward real-world deployment of machine learning for health care: external validation, continual monitoring, and randomized clinical trials. Health Care Sc. (2024) 3:360. 10.1002/HCS2.114 39479276 PMC11520244

[45] Hofer IS Burns M Kendale S Wanderer JP. Realistically integrating machine learning into clinical practice: a road map of opportunities, challenges, and a potential future. Anesth Analg. (2020) 130:1115. 10.1213/ANE.0000000000004575 32287118 PMC7584400

[46] Chang W-C Dai L Xu T. Machine learning approaches to clinical risk prediction: multi-scale temporal alignment in electronic health records (2025).

[47] Wang J Luo J Ye M Wang X Zhong Y Chang A Recent advances in predictive modeling with electronic health records. IJCAI. (2024) 2024:8272. 10.24963/IJCAI.2024/914 40248670 PMC12005588

[48] Carrasco-Ribelles LA Llanes-Jurado J Gallego-Moll C Cabrera-Bean M Monteagudo-Zaragoza M Violán C Prediction models using artificial intelligence and longitudinal data from electronic health records: a systematic methodological review. J Am Med Inform Assoc. (2023) 30:2072. 10.1093/JAMIA/OCAD168 37659105 PMC10654870

[49] Liu T Krentz A Lu L Curcin V. Machine learning based prediction models for cardiovascular disease risk using electronic health records data: systematic review and meta-analysis. Eur Heart J Digit Health. (2025) 6:7–22. 10.1093/EHJDH/ZTAE080 39846062 PMC11750195

[50] Prieto-Merino D Mulick A Armstrong C Hoult H Fawcett S Eliasson L Estimating proportion of days covered (PDC) using real-world online medicine suppliers’ datasets. J Pharm Policy Pract. (2021) 14:113. 10.1186/S40545-021-00385-W 34965882 PMC8715592

[51] Raebel MA Carroll NM Ellis JL Schroeder EB Bayliss EA. Importance of including early non-adherence in estimations of medication adherence. Ann Pharmacother. (2011) 45:1053. 10.1345/APH.1Q146 21852598 PMC5490834

[52] Chang HY Kan HJ Shermock KM Caleb Alexander G Weiner JP Kharrazi H. Integrating E-prescribing and pharmacy claims data for predictive modeling: comparing costs and utilization of health plan members who fill their initial medications with those who do not. J Manag Care Spec Pharm. (2020) 26:1282–90. 10.18553/JMCP.2020.26.10.1282 32996394 PMC10391092

[53] Arnet I Abraham I Messerli M Hersberger KE. A method for calculating adherence to polypharmacy from dispensing data records. Int J Clin Pharm. (2013) 36:192. 10.1007/S11096-013-9891-8 24293284 PMC3890044

[54] Hofmeyer BA Look KA Hager DR. Refill-based medication use quality measures in kidney transplant recipients: examination of proportion of days covered and medication possession ratio. J Manag Care Spec Pharm. (2018) 24:367–72. 10.18553/JMCP.2018.24.4.367 29578851 PMC10398127

[55] Isgut M Hornback A Bao H Sun Y Choi K Anderson BJ Greater value add from electronic health records than polygenic risk scores for predicting myocardial infarction in machine learning. Commun Med. (2025) 5:450. 10.1038/s43856-025-01138-5 41184489 PMC12583447

[56] Rasu RS Hunt SL Dai J Cui H Phadnis MA Jain N. Accurate medication adherence measurement using administrative data for frequently hospitalized patients. Hosp Pharm. (2021) 56:451–61. 10.1177/0018578720918550 34720145 PMC8554601

[57] Ambati SKN. SAS Meets machine learning: an adaptive framework for healthcare data fusion. Int J Sci Res Comput Sci Eng Inf Technol. (2025) 11:1456–65. 10.32628/CSEIT251112151

[58] Jarmakovica A. Machine learning-based strategies for improving healthcare data quality: an evaluation of accuracy, completeness, and reusability. Front Artif Intell. (2025) 8:1621514. 10.3389/FRAI.2025.1621514/BIBTEX 40761812 PMC12319021

[59] Ren W Liu Z Wu Y Zhang Z Hong S Liu H. Moving beyond medical statistics: a systematic review on missing data handling in electronic health records. Health Data Sci. (2024) 4:0176. 10.34133/HDS.0176 39635227 PMC11615160

[60] Serhal S Armour C Billot L Krass I Emmerton L Saini B Integrating pharmacy and registry data strengthens clinical assessments of patient adherence. Front Pharmacol. (2022) 13:869162. 10.3389/FPHAR.2022.869162/FULL 35401235 PMC8990834

[61] Maletzky A Böck C Tschoellitsch T Roland T Ludwig H Thumfart S Lifting hospital electronic health record data treasures: challenges and opportunities. JMIR Med Inform. (2022) 10:e38557. 10.2196/38557 36269654 PMC9636533

[62] Ghassemi M Naumann T Schulam P Beam AL Chen IY Ranganath R. A review of challenges and opportunities in machine learning for health. AMIA Jt Summits Transl Sci Proc. (2020) 2020:191–200. https://www.ncbi.nlm.nih.gov/pmc/articles/PMC7233077/PMC7233077 32477638

[63] Scarton L Nelson T Yao Y Segal R Donahoo WT Goins RT Medication adherence and cardiometabolic control indicators among American Indian adults receiving tribal health services: protocol for a longitudinal electronic health records study. JMIR Res Protoc. (2022) 11:e39193. 10.2196/39193 36279173 PMC9641513

[64] Balaji P Mohitha IK Prashanthini K Sivaprakash K Rithick P Kasthuri S. Optimizing medication compliance through machine learning and IoT for personalized health management. In Proceedings of the 9 International Conference on Communication and Electronics Systems, ICCES 2024. (2024). p. 1111–7.

[65] O’Brien MK Hohl K Lieber RL Jayaraman A. Automate, illuminate, predict: a universal framework for integrating wearable sensors in healthcare. Digit Biomark. (2024) 8:149. 10.1159/000540492 39473803 PMC11521435

[66] Iino H Kizaki H Imai S Hori S. Medication management initiatives using wearable devices: scoping review. JMIR Hum Factors. (2024) 11:e57652. 10.2196/57652 39602520 PMC11612519

[67] Mukherjee S Suleman S Pilloton R Narang J Rani K. State of the art in smart portable, wearable, ingestible and implantable devices for health Status monitoring and disease management. Sensors. (2022) 22:4228. 10.3390/S22114228 35684847 PMC9185336

[68] Rough K Dai AM Zhang K Xue Y Vardoulakis LM Cui C Predicting inpatient medication orders from electronic health record data. Clin Pharmacol Ther. (2020) 108:145. 10.1002/CPT.1826 32141068 PMC7325318

[69] Zhang Y Golbus JR Wittrup E Aaronson KD Najarian K. Enhancing heart failure treatment decisions: interpretable machine learning models for advanced therapy eligibility prediction using EHR data. BMC Med Inform Decis Mak. (2024) 24:53. 10.1186/S12911-024-02453-Y 38355512 PMC10868035

[70] Coutinho-Almeida J Saez C Correia R Rodrigues PP. Development and initial validation of a data quality evaluation tool in obstetrics real-world data through HL7-FHIR interoperable Bayesian networks and expert rules. JAMIA Open. (2024) 7:ooae062. 10.1093/JAMIAOPEN/OOAE062 39070966 PMC11283181

[71] Endebu T Taye G Deressa W. Development of a machine learning prediction model for loss to follow-up in HIV care using routine electronic medical records in a low-resource setting. BMC Med Inform Decis Mak. (2025) 25:192. 10.1186/S12911-025-03030-7 40389908 PMC12090508

[72] Ogbechie MD Walker CF Lee MT Gana AA Oduola A Idemudia A Predicting treatment interruption among people living with HIV in Nigeria: machine learning approach. JMIR AI. (2023) 2:e44432. 10.2196/44432 38875546 PMC11041440

[73] Lu ZH Yang M Pan CH Zheng PY Zhang SX. Multi-modal deep learning based on multi-dimensional and multi-level temporal data can enhance the prognostic prediction for multi-drug resistant pulmonary tuberculosis patients. Sci One Health. (2022) 1:100004. 10.1016/J.SOH.2022.100004 39076608 PMC11262254

[74] Anley DT Akalu TY Dessie AM Anteneh RM Zemene MA Bayih WA Prognostication of treatment non-compliance among patients with multidrug-resistant tuberculosis in the course of their follow-up: a logistic regression–based machine learning algorithm. Front Digit Health. (2023) 5:1165222. 10.3389/FDGTH.2023.1165222 37228302 PMC10203954

[75] Sambarey A Smith K Chung C Arora HS Yang Z Agarwal PP Integrative analysis of multimodal patient data identifies personalized predictors of tuberculosis treatment prognosis. iScience. (2024) 27:109025. 10.1016/J.ISCI.2024.109025 38357663 PMC10865408

[76] Wang Z Guo Z Wang W Zhang Q Song S Xue Y Prediction of tuberculosis treatment outcomes using biochemical makers with machine learning. BMC Infect Dis. (2025) 25:229. 10.1186/S12879-025-10609-Y 39962412 PMC11834319

[77] Chen YL Nguyen PA Chien CH Hsu MH Liou DM Yang HC. Machine learning-based prediction of medication refill adherence among first-time insulin users with type 2 diabetes. Diabetes Res Clin Pract. (2024) 207:111033. 10.1016/j.diabres.2023.111033 38049037

[78] Lavikainen P Chandra G Siirtola P Tamminen S Ihalapathirana AT Röning J Data-driven identification of long-term glycemia clusters and their individualized predictors in Finnish patients with type 2 diabetes. Clin Epidemiol. (2023) 15:13–29. 10.2147/CLEP.S380828 36636731 PMC9829833

[79] Chaikijurajai T Laffin LJ Wilson Tang WH. Artificial intelligence and hypertension: recent advances and future outlook. Am J Hypertens. (2020) 33:967. 10.1093/AJH/HPAA102 32615586 PMC7608522

[80] Mroz T Griffin M Cartabuke R Laffin L Russo-Alvarez G Thomas G Predicting hypertension control using machine learning. PLoS One. (2024) 19:e0299932. 10.1371/JOURNAL.PONE.0299932 38507433 PMC10954144

[81] Byeon H. Determinants of blood pressure control in hypertensive individuals using histogram-based gradient boosting: findings from 1114 male workers in South Korea. J Mens Health. (2024) 20:47–55. 10.22514/JOMH.2024.148/HTM

[82] Hae H Kang S-J Kim TO Lee H Lee S-W Kim Y-H Machine learning-based prediction of post-treatment ambulatory blood pressure in patients with hypertension. Blood Press. (2023) 32:2209674. 10.1080/08037051.2023.2209674 37211803

[83] Hu Y Huerta J Cordella N Mishuris RG Paschalidis IC. Personalized hypertension treatment recommendations by a data-driven model. BMC Med Inform Decis Mak. (2023) 23:44. 10.1186/S12911-023-02137-Z 36859187 PMC9979505

[84] Dennis JM Young KG Cardoso P Güdemann LM McGovern AP Farmer A A five-drug class model using routinely available clinical features to optimise prescribing in type 2 diabetes: a prediction model development and validation study. Lancet. (2025) 405:701–14. 10.1016/S0140-6736(24)02617-5 40020703

[85] Pei X Du X Liu D Li X Wu Y. Nomogram model for predicting medication adherence in patients with various mental disorders based on the dryad database. BMJ Open. (2024) 14:e087312. 10.1136/BMJOPEN-2024-087312 39542487 PMC11575275

[86] Tay JL Htun KK Sim K. Prediction of clinical outcomes in psychotic disorders using artificial intelligence methods: a scoping review. Brain Sci. (2024) 14:878. 10.3390/BRAINSCI14090878/S1 39335374 PMC11430394

[87] Beaudoin M Potvin S Hudon A Giguère C-E Dumais A. Prediction of quality of life in schizophrenia using machine learning models on data from clinical antipsychotic trials of intervention effectiveness (CATIE) schizophrenia trial. Eur Psychiatry. (2021) 64:S157. 10.1192/J.EURPSY.2021.423 PMC8938459 35314708

[88] de Nijs J Burger TJ Janssen RJ Kia SM van Opstal DPJ de Koning MB Individualized prediction of three- and six-year outcomes of psychosis in a longitudinal multicenter study: a machine learning approach. NPJ Schizophr. (2021) 7:34. 10.1038/S41537-021-00162-3 34215752 PMC8253813

[89] Wong TY Luo H Tang J Moore TM Gur RC Suen YN Development of an individualized risk calculator of treatment resistance in patients with first-episode psychosis (TRipCal) using automated machine learning: a 12-year follow-up study with clozapine prescription as a proxy indicator. Transl Psychiatry. (2024) 14:50. 10.1038/S41398-024-02754-W 38253484 PMC10803337

[90] Chen J Jiang Y Li Z Zhang M Liu L Li A Predictive machine learning models for anticipating loss to follow-up in tuberculosis patients throughout anti-TB treatment journey. Sci Rep. (2024) 14:24685. 10.1038/s41598-024-74942-z 39433802 PMC11494039

[91] Kassaw EA Sendekie AK Enyew BM Abate BB. Machine learning applications to classify and monitor medication adherence in patients with type 2 diabetes in Ethiopia. Front Endocrinol. (2025) 16:1486350. 10.3389/FENDO.2025.1486350/BIBTEX PMC11965118 40182636

[92] Li M Lu X Yang HB Yuan R Yang Y Tong R Development and assessment of novel machine learning models to predict medication non-adherence risks in type 2 diabetics. Front Public Health. (2022) 10:1000622. 10.3389/FPUBH.2022.1000622 36466490 PMC9714465

[93] Cabot JH Ross EG. Evaluating prediction model performance. Surgery. (2023) 174:723. 10.1016/J.SURG.2023.05.023 37419761 PMC10529246

[94] Hu Q Chen Y Zou D He Z Xu T. Predicting adverse drug event using machine learning based on electronic health records: a systematic review and meta-analysis. Front Pharmacol. (2024) 15:1497397. 10.3389/FPHAR.2024.1497397/BIBTEX 39605909 PMC11600142

[95] Piovani D Sokou R Tsantes AG Vitello AS Bonovas S. Optimizing clinical decision making with decision curve analysis: insights for clinical investigators. Healthcare. (2023) 11:2244. 10.3390/healthcare11162244 37628442 PMC10454914

[96] Ehrmann DE Joshi S Goodfellow SD Mazwi ML Eytan D. Making machine learning matter to clinicians: model actionability in medical decision-making. NPJ Digit Med. (2023) 6:7. 10.1038/S41746-023-00753-7 36690689 PMC9871014

[97] Duan X Liu Y Shang Y Lu X Zhou Y Liu L Development and validation of an interpretable machine learning model for acute radiation dermatitis in breast cancer. Front Oncol. (2025) 15:1663293. 10.3389/FONC.2025.1663293/BIBTEX 41179669 PMC12575144

[98] Li J Zou L Ma H Zhao J Wang C Li J Interpretable machine learning based on CT-derived extracellular volume fraction to predict pathological grading of hepatocellular carcinoma. Abdominal Radiology. (2024) 49:3383–96. 10.1007/S00261-024-04313-9 38703190

[99] Chandrika M Kiran R Vaidya P Kodnad R. Evaluation of machine learning models for cardiovascular risk assessment. In 2nd International Conference on Intelligent Cyber Physical Systems and Internet of Things, ICoICI 2024. (2024). p. 993–6.

[100] Schwabe D Becker K Seyferth M Klaß A Schaeffter T. The METRIC-framework for assessing data quality for trustworthy AI in medicine: a systematic review. NPJ Digit Med. (2024) 7:203. 10.1038/S41746-024-01196-4 39097662 PMC11297942

[101] Mišić VV Rajaram K Gabel E. A simulation-based evaluation of machine learning models for clinical decision support: application and analysis using hospital readmission. NPJ Digit Med. (2021) 4:98. 10.1038/S41746-021-00468-7 34127786 PMC8203794

[102] Dixon D Sattar H Moros N Kesireddy SR Ahsan H Lakkimsetti M Unveiling the influence of AI predictive analytics on patient outcomes: a comprehensive narrative review. Cureus. (2024) 16:e59954. 10.7759/CUREUS.59954 38854327 PMC11161909

[103] Panch T Mattie H Atun R. Artificial intelligence and algorithmic bias: implications for health systems. J Glob Health. (2019) 9:020318. 10.7189/JOGH.09.020318 31788229 PMC6875681

[104] Gu T Pan W Yu J Ji G Meng X Wang Y Mitigating bias in AI mortality predictions for minority populations: a transfer learning approach. BMC Med Inform Decis Mak. (2025) 25:30. 10.1186/S12911-025-02862-7 39825353 PMC11742213

[105] Wu H Zhu Y Shi W Tong L Wang MD. Fairness artificial intelligence in clinical decision support: mitigating effect of health disparity. IEEE J Biomed Health Inform. (2025) 29:815–23. 10.1109/JBHI.2024.3513398

[106] Goktas P Grzybowski A. Shaping the future of healthcare: ethical clinical challenges and pathways to trustworthy AI. J Clin Med. (2025) 14:1605. 10.3390/JCM14051605 40095575 PMC11900311

[107] Mwogosi A. Ethical and privacy challenges of integrating generative AI into EHR systems in Tanzania: a scoping review with a policy perspective. Digit Health. (2025) 11:20552076251344385. 10.1177/20552076251344385 40400763 PMC12093014

[108] Tiffin N George A Lefevre AE. How to use relevant data for maximal benefit with minimal risk: digital health data governance to protect vulnerable populations in low-income and middle-income countries. BMJ Glob Health. (2019) 4:e001395. 10.1136/BMJGH-2019-001395 31139457 PMC6509603

[109] Arjanto P Makulua IJ Sampe PD Huliselan N Ellis R. Augmenting human connection: a systematic review of artificial intelligence in counseling practices, ethics, and cultural adaptation. J Bimbing Konseling Pandohop. (2026) 6:1–15. 10.37304/PANDOHOP.V6I1.20310

[110] Goodman KW Litewka SG Malpani R Pujari S Reis AA. Global health and big data: the WHO’s artificial intelligence guidance. S Afr J Sci. (2023) 119:14725. 10.17159/SAJS.2023/14725 39328373 PMC11426405

[111] Dolatkhah Laein G. Global perspectives on governing healthcare AI: prioritising safety, equity and collaboration. BMJ Lead. (2024) 9:e000904. 10.1136/LEADER-2023-000904 PMC12038151 38806230

[112] Yang YT Ricciardi R. Regulating AI in nursing and healthcare: ensuring safety, equity, and accessibility in the era of federal innovation policy. Policy Polit Nurs Pract. (2025) 27(1):17–25. 10.1177/15271544251381228 41032683

[113] Rahimi AK Pienaar O Ghadimi M Canfell OJ Pole JD Shrapnel S Implementing AI in hospitals to achieve a learning health system: systematic review of current enablers and barriers. J Med Internet Res. (2024) 26:e49655. 10.2196/49655 39094106 PMC11329852

[114] Liang AS Amrollahi F Jiang Y Corbin CK Kim GY Mui D SmartAlert: implementing machine learning-driven clinical decision support for inpatient lab utilization reduction (2025). 10.1056/aics2501086 PMC13367503 42453199

[115] Wong A Roslan NL McDonald R Noor J Hutchings S D’Costa P Clinical obstacles to machine-learning POCUS adoption and system-wide AI implementation (the COMPASS-AI survey). Ultrasound J. (2025) 17:32. 10.1186/S13089-025-00436-2 40608159 PMC12229359

[116] Gunlicks-Stoessel M Liu Y Parkhill C Morrell N Choy-Brown M Mehus C Adolescent, parent, and provider attitudes toward a machine learning based clinical decision support system for selecting treatment for youth depression. BMC Med Inform Decis Mak. (2023) 24:4. 10.1186/S12911-023-02410-1 PMC10759496 38167319

[117] Nguyen K Wilson DL Diiulio J Hall B Militello L Gellad WF Design and development of a machine-learning-driven opioid overdose risk prediction tool integrated in electronic health records in primary care settings. Bioelectron Med. (2024) 10:24. 10.1186/S42234-024-00156-3 39420438 PMC11488086

[118] Pennestrì F Cabitza F Picerno N Banfi G. Sharing reliable information worldwide: healthcare strategies based on artificial intelligence need external validation. Position paper. BMC Med Inform Decis Mak. (2025) 25:56. 10.1186/S12911-025-02883-2 39905337 PMC11796012

[119] Chen J Hou D Song Y. Development and multi-database validation of interpretable machine learning models for predicting in-hospital mortality in pneumonia patients: a comprehensive analysis across four healthcare systems. Respir Res. (2025) 26:279. 10.1186/S12931-025-03348-W/FIGURES/6 41029388 PMC12486837

[120] Valan B Prakash A Ratliff W Gao M Muthya S Thomas A Evaluating sepsis watch generalizability through multisite external validation of a sepsis machine learning model. NPJ Digit Med. (2025) 8:350. 10.1038/s41746-025-01664-5 40500319 PMC12159134

[121] Reps JM Williams RD You SC Falconer T Minty E Callahan A Feasibility and evaluation of a large-scale external validation approach for patient-level prediction in an international data network: validation of models predicting stroke in female patients newly diagnosed with atrial fibrillation. BMC Med Res Methodol. (2020) 20:102. 10.1186/S12874-020-00991-3 32375693 PMC7201646

[122] Anteneh A Belachew M Birhanu S Meseret M. Predicting cardiovascular disease among diabetic patients in Ethiopia using machine learning models: evidence from Ethiopian public health institute data (2024/2025). BMC Public Health. (2025) 25:4114. 10.1186/S12889-025-24850-2 41286785 PMC12642091

